# Development of the equine hindgut microbiome in semi-feral and domestic conventionally-managed foals

**DOI:** 10.1186/s42523-020-00060-6

**Published:** 2020-11-23

**Authors:** Meredith K. Tavenner, Sue M. McDonnell, Amy S. Biddle

**Affiliations:** 1 Department of Animal and Food Sciences, University of Delaware, College of Agriculture and Natural Resources, Newark, DE 19711 USA; 2 Havemeyer Equine Behavior Laboratory, New Bolton Center, University of Pennsylvania School of Veterinary Medicine, Kennett Square, PA 19348 USA

**Keywords:** Microbial diversity, Gut microbiome acquisition, Equine management

## Abstract

**Background:**

Early development of the gut microbiome is an essential part of neonate health in animals. It is unclear whether the acquisition of gut microbes is different between domesticated animals and their wild counterparts. In this study, fecal samples from ten domestic conventionally managed (DCM) Standardbred and ten semi-feral managed (SFM) Shetland-type pony foals and dams were compared using 16S rRNA sequencing to identify differences in the development of the foal hindgut microbiome related to time and management.

**Results:**

Gut microbiome diversity of dams was lower than foals overall and within groups, and foals from both groups at Week 1 had less diverse gut microbiomes than subsequent weeks. The core microbiomes of SFM dams and foals had more taxa overall, and greater numbers of taxa within species groups when compared to DCM dams and foals. The gut microbiomes of SFM foals demonstrated enhanced diversity of key groups: Verrucomicrobia (RFP12), Ruminococcaceae, *Fusobacterium* spp., and *Bacteroides* spp., based on age and management. Lactic acid bacteria *Lactobacillus* spp. and other Lactobacillaceae genera were enriched only in DCM foals, specifically during their second and third week of life. Predicted microbiome functions estimated computationally suggested that SFM foals had higher mean sequence counts for taxa contributing to the digestion of lipids, simple and complex carbohydrates, and protein. DCM foal microbiomes were more similar to their dams in week five and six than were SFM foals at the same age.

**Conclusions:**

This study demonstrates the impact of management on the development of the foal gut microbiome in the first 6 weeks of life. The higher numbers of taxa within and between bacterial groups found in SFM dams and foals suggests more diversity and functional redundancy in their gut microbiomes, which could lend greater stability and resiliency to these communities. The colonization of lactic acid bacteria in the early life of DCM foals suggests enrichment in response to the availability of dams’ feed. Thus, management type is an important driver of gut microbiome establishment on horses, and we may look to semi-feral horses for guidance in defining a healthy gut microbiome for domestic horses.

**Supplementary Information:**

**Supplementary information** accompanies this paper at 10.1186/s42523-020-00060-6.

## Background

The gut microbiome is important for immune response, gastrointestinal tract health, endocrine system functioning, behavior, and even cognitive function in both humans and animals [1–6]. In humans, gut dysbiosis has been linked to many conditions, including obesity, autism spectrum disorders, diabetes, colorectal cancer, inflammatory bowel diseases as well as diseases caused by pathogenic bacteria [7–10]. In the horse, common gastrointestinal disorders have been associated with gut dysbiosis, including starch-induced laminitis, colitis, diarrhea and gastric ulcers [11–15]. These abnormalities have been correlated with differences in microbial diversity and abundances when compared to healthy horses.

The early development of the gut microbiome is an essential part of immune system training and maintenance of a healthy neonate. Failure to establish healthy commensal interactions in early development can result in chronic inflammation and autoimmune issues [16, 17]. Studies specifically focusing on the early development of the equine gut have found that the foal’s bacterial community stabilizes to that similar to an adult horse at approximately 1 to 2 months of age [18, 19]. A comparison of the gut microbiomes of 11 mare-foal pairs showed a higher abundance of Acidobacteria in newborn foals than mares, a higher abundance of Fibrobacteres and Spirochaetes in foals aged 121–240 days than mares and a lower abundance of Chlamydiae in mares than foals aged 31–60 days [19]. Another study using 16S rDNA sequencing to characterize the microbiomes of foals in the first 10 days of life and their respective Standardbred dams, reported that the initial colonization of foals’ gut microbiota (from the meconium) reflected bacteria found in the dams’ milk, including *Enterococcus* spp*.* and Enterobacteriaceae [20]. By day three, the foals’ gut bacterial communities were similar to that of their dams’, with the acquisition of fiber fermenting microorganisms. The impact of management, and specific drivers on the early development of the foal microbiome are unclear.

Short-term studies of the foal gut microbiota have focused on effects of diarrhea, *Rhodococcus equi* pneumonia vaccination, weaning, and probiotic supplementation, identifying specific pathogenic bacteria or determining changes in the diversity of the foals’ microbiomes [18, 21, 22]. Development of the foal microbiome is suggested to be established prior to weaning since no difference in gut microbiome species diversity or community membership were found between foals experiencing gradual and abrupt weaning [18], and foals’ microbiomes were not significantly different than their dams’ beginning at 1 month of age [19]. In this study, we surveyed the gut microbiome of normal foals with respect to their dams for the first 6 weeks of life in order to map the acquisition of bacterial community members and inferred functions.

Management factors such as grazing access, exercise, social interaction and diet contribute to equine health. Horses are naturally adapted to be continuous grazers, however grain-based feed is often added to the diets of domestic horses to meet their energy requirements, and domestically managed horses often experience intermittent fasting. Horses that are able to continuously graze secrete more saliva, which buffers the acidity of their stomach contents. Compared with semi-feral horses and ponies, domestically managed equines have greater incidence of gastric ulcers [23] than feral horses.

Comparisons of the gut microbiomes of domestic and feral or semi-feral horses have shown differences in diversity and community structure. When compared to domestic horses living in adjacent grassland, feral Przewalski’s horses had a distinct and more diverse bacterial community [24]. Feral Przewalski’s horses had a higher abundance of the orders Clostridiales, Bacteroidales and Erysipelotrichales, while domestic horses had a higher abundance of Spirochaetales. Additionally, the feral horses less than a year of age had a less diverse and more compositionally distinct microbial community than those older than 1 year old [24]. Bacterial 16S rDNA surveys of fecal samples from Hokkaido native horses and light horses observed that native horses had a more diverse microbiome than light horses as well as a higher abundance of *Fibrobacter succinogenes* [25]. A specific cluster of bacteria related to cellulolytic bacteria were only found in native horses while one related to soluble sugar-utilizing species were only found in light horses [25].

The objective of this study was to identify differences in the patterns of acquisition and inferred function between the gut microbiomes of semi-feral and domestically raised foals. Semi-feral management mimics the horses’ “natural” state. Therefore the comparison made in this study informs management practices such as access to pasture, grain, and/or other horses with the potential to impact microbiome development at the earliest ages of life.

## Results

### Microbial composition for dams and foals

Samples were taken weekly for the first 6 weeks of life from 20 foals (n_samples_ = 116) and 20 dams (n_samples_ = 20) for a total of 136. There were a total of 81,365 observed operational taxonomic units (OTUs) from foal and dam samples and a total of 3,887,277 sequence counts (mean ± s.d = 28,582.92 ± 16,448.23; range = 3469-69,307; median = 26,783.5). Average read length was 409.9628 +/− 2.552. OTUs with fewer than three reads were removed from analysis. The sequencing blank was found to have 86 OTUs and a total of 7996 sequences. Three OTUs found to be in common with the sequencing blank and 100% of the samples were subtracted from further analysis: Unassigned, Other, Ruminococcaceae; g__, and g__Bacteroides. (Additional file 1).

**Fig. 1:**
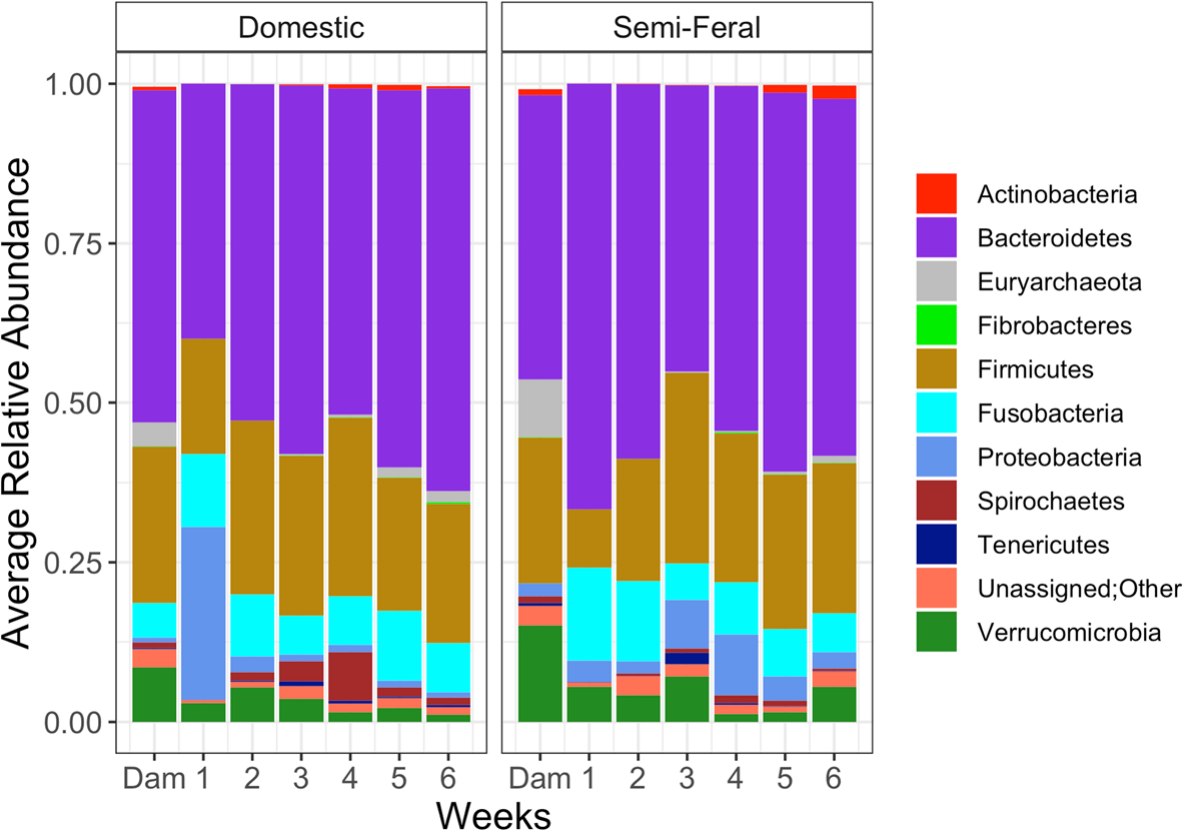
Comparison of the microbiomes semi-feral and domestic dams and foals at different age groups at the phylum level. Low abundance phyla (represented in fewer than 60% of samples) are not shown: Armatimonadetes, Chlamydiae, Cyanobacteria, Elusimicrobia, Lentisphaerae, Planctomycetes, Synergistetes, WPS-2

OTUs were classified into 19 phyla (Fig. 1). The most abundant phylum present was Bacteroidetes followed by Firmicutes in both foals and dams. The average abundance of Bacteroidetes in foals and dams was 55.2 and 48.3%, respectively, and the average abundance of Firmicutes in foals and dams was 22.5 and 23.7%, respectively.

**Fig. 2:**
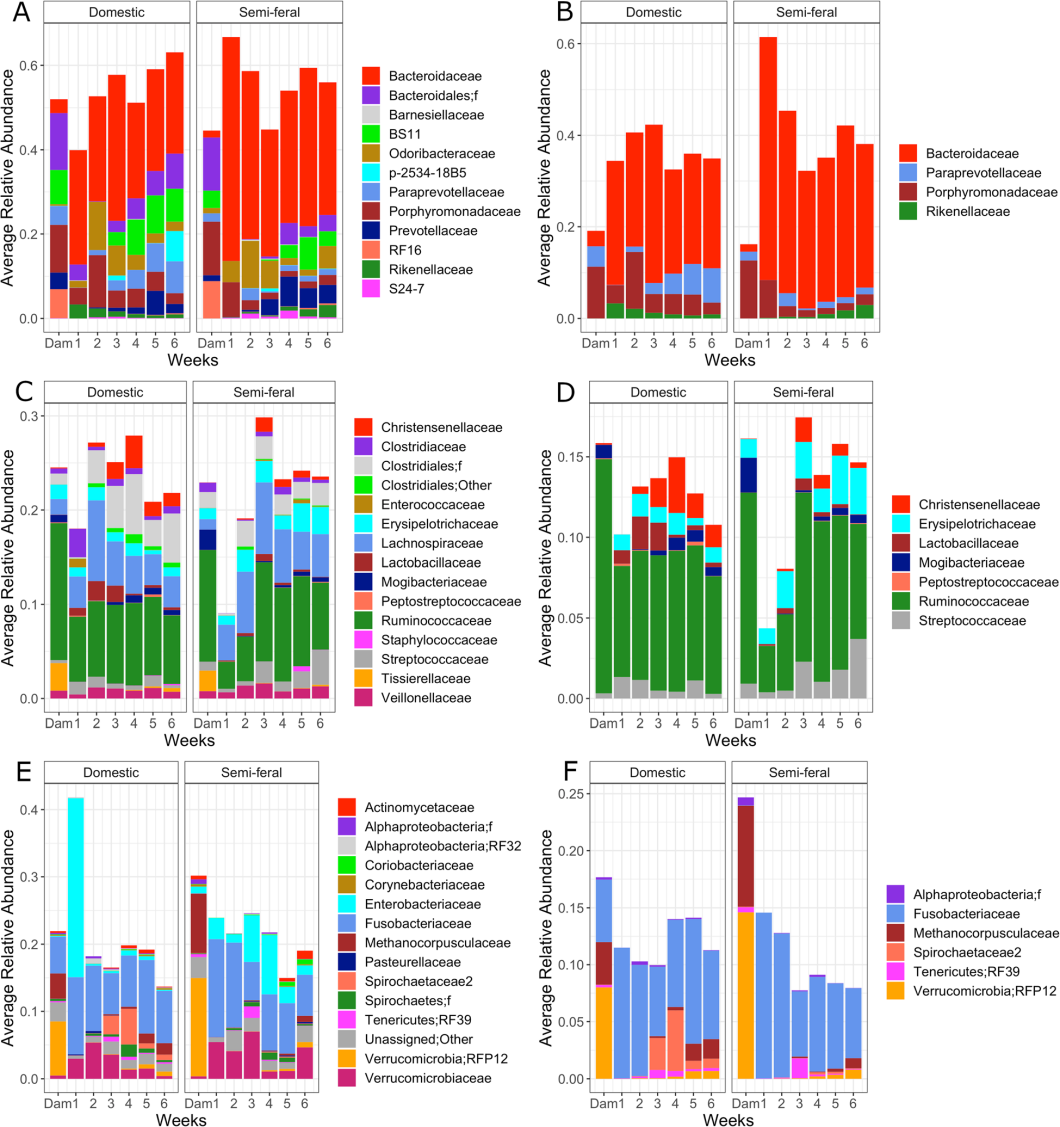
**a** Average relative abundances of Bacteroidetes by family for DCM and SFM dams and foals across the time course. Families with less than 0.01% relative abundance for all samples are not shown. **b** Bacteroidetes families that were significantly different between DCM and SFM dams and foals (*p* < 0.05, pair-wise t-tests). **c** Average relative abundances of Firmicutes by family for DCM and SFM dams and foals across the time course. Families with less than 0.01% relative abundance for all samples are not shown. **d** Firmicutes families that were significantly different between DCM and SFM dams and foals (*p* < 0.05, pair-wise t-tests). **e** Average relative abundances of non-Firmicutes/ Bacteroidetes by family for DCM and SFM dams and foals across the time course. Families with less than 0.01% relative abundance for all samples are not shown. **f** Families that were significantly different between DCM and SFM dams and foals (*p* < 0.05, pair-wise t-tests). Undefined families in an order are identified as f

At the family level, four Bacteroidetes families were found to be significantly different between DCM and SFM dams and foals across the time course: Bacteroidaceae was enriched in SFM groups, while Paraprevotellaceae, Porphyromonadaceae, and Rikenellaceae were more abundant in DCM groups (Fig. 2a and b). Seven Firmicutes families were found to be significantly different between DCM and SFM dams and foals across the time course: Mogibacteriaceae, Streptococcaceae, and Erysipelotrichaceae were enriched in SFM groups, while Christensenellaceae, Lactobacillaceae, and Peptostreptococcaceae were more abundant in DCM groups (Fig. 2c and d). Six families in other phyla were found to be significantly different between DCM and SFM dams and foals across the time course: Fusobacteriaceae and a family of Tenericutes (RF39) were enriched in SFM foals, a family of Verrucomicrobia (RFP12) and an Alphaproteobacteria family were more abundant in SFM dams, while Methanocorpusculaceae and a family of Spirochaetes were more abundant in DCM groups (Fig. 2e and f).

### Effect of breed on Horse’s hindgut microbiome

OTUs picked using the combined sequence files from the dams in this study and the adult ponies and Standardbred horses in the EMP database [26] revealed no significant taxa differences with respect to breed (Kruskal-Wallis or nonparametric t-test, corrected *p* > 0.05), however there were 570 taxa were found to be different between the groups with respect to study [Additional files 2 and 3]. Alpha diversity differences between the EMP horses and the dams in the current study highlight that while factors inherent to each study impact microbiome composition, breed does not appear to be a major driver. [Additional file 4].

**Fig. 3:**
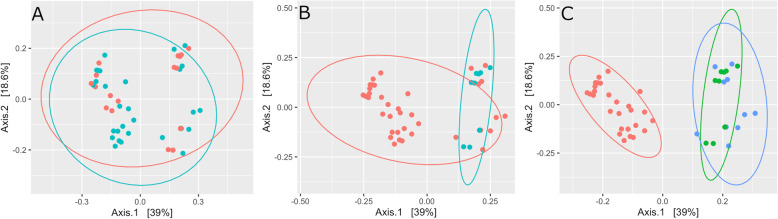
PCoA plot of the relationships between the beta diversity of the DCM and SFM dam microbiota and comparative samples from the EMP database using Bray-Curtis Dissimilarity. Ellipsoids representing a 95% confidence interval. Color by: **a.** Breed: Pony (red), Standardbred (blue), **b.** Management: Domestic (red), Semiferal (blue), **c**. Farm: EMP (red), Winback Farm (blue), New Bolton Center (green)

Clustering of samples by principle coordinate analysis (PCoA) of Bray-Curtis Dissimilarity point to significant beta diversity differences based on management and study (Fig. 3). The Goods coverage of the EMP samples was 85.74%.

### Core microbiomes of SFM and DCM dams and foals

**Fig. 4:**
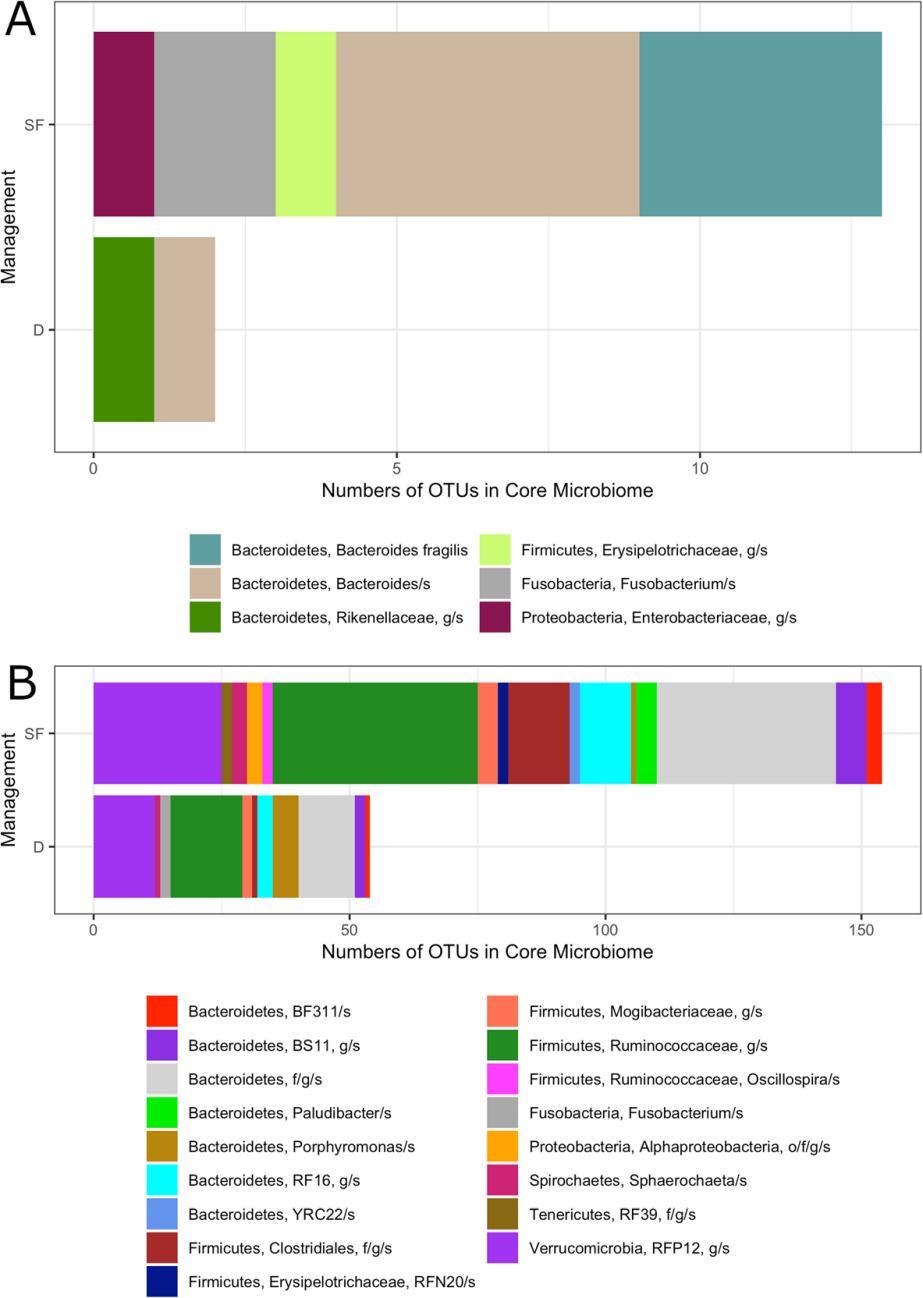
Numbers of OTUs by taxa group in the core microbiomes (present in 95% or more of samples in each group) of SFM (SF) and DCM (D) managed horses. **a.** Foals, **b.** Dams

The core microbiomes of SFM and DCM foals and dams, defined as OTUs present in 95% or more of samples in each group, were different in terms of composition and numbers of OTUs comprising each taxon (Fig. 4). Overall, SFM foals and dams had higher numbers of taxa in their core microbiomes, and more OTUs in almost every group. The core microbiome of SFM foals was comprised of five taxa, only one of which, *Bacteroides* spp., was shared with DCM foals (Fig. 4a). For this shared taxa, the SFM core microbiome featured five OTUs, while the DCM core microbiome had one. Besides *Bacteroides* spp., the core microbiome of DCM foals contained a *Rikenellaceae* spp., which was not shared with the SFM foals, and the SFM foal core microbiome included four unique taxa groups: *Bacteroides fragilis*, *Enterobacteriaceae* spp., *Erysipelotrichaceae* spp., and *Fusobacterium* spp. (Fig. 4a). The core microbiome of SFM dams featured 154 OTUs in 16 taxa groups, while that of DCM dams had 54 OTUs in 11 taxa groups (Fig. 4b). Unique taxa groups found in the SFM core microbiome for dams included: *Paulibacter* spp., *YRC22* spp., *RFN20* spp., *Oscillospira* spp., *Alphaproteobacteria* spp., and *RFP12* spp. Only one taxon, *Fusobacterium* spp. was unique to the DCM core microbiome for dams. This taxon was found in the core microbiome of SFM foals, but not in that of DCM foals, and was the only taxa group that overlapped between the dams and foals regardless of management.

### Alpha and Beta diversity

**Fig. 5:**
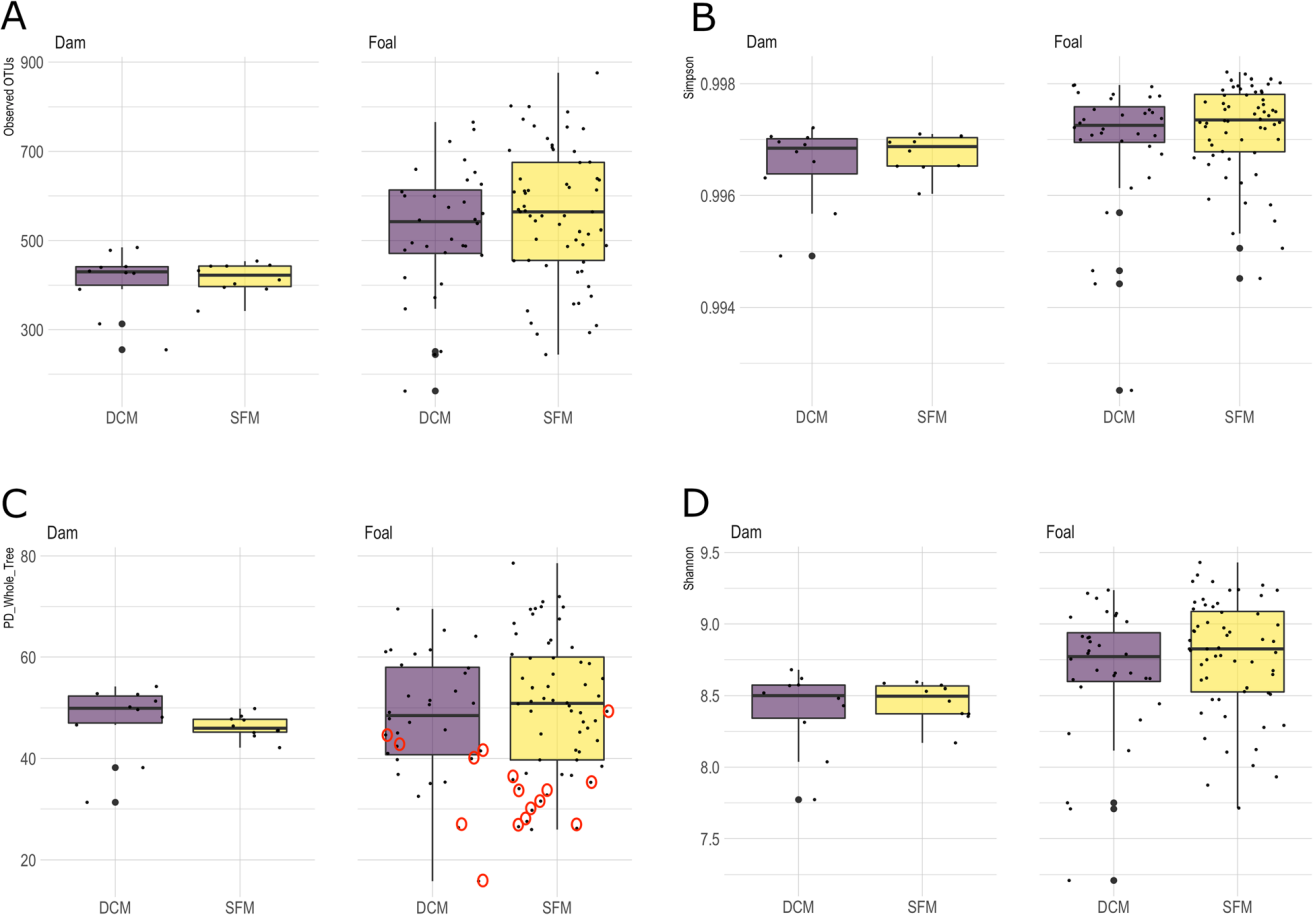
Alpha diversity of gut microbiome communities for DCM and SFM dams and foals. **a.** Observed OTUs, **b.** Simpson, **c.** PD_Whole_Tree, (Week 1 samples circled in red) **d.** Shannon

**Fig. 6:**
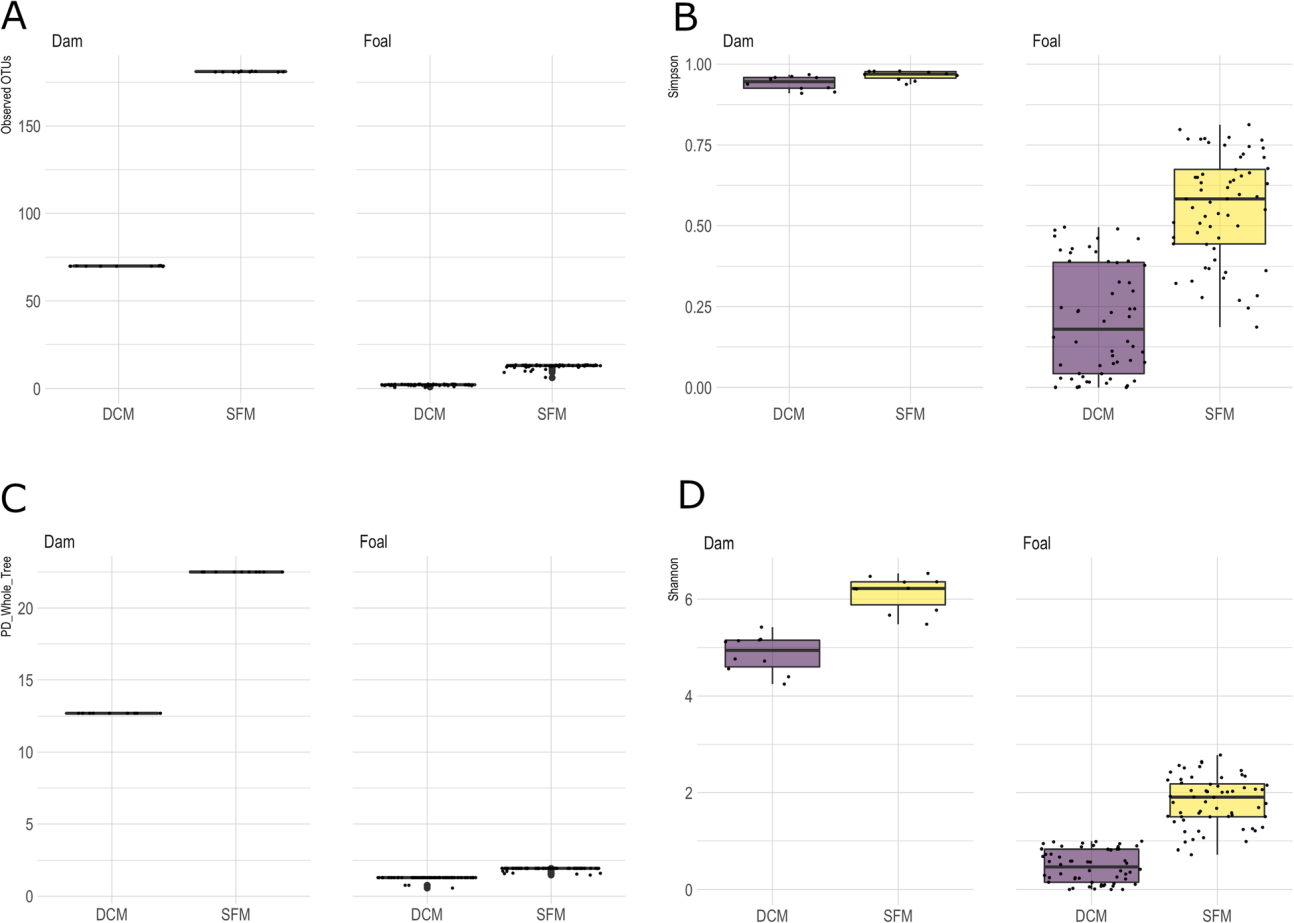
Alpha diversity of core microbiome communities of DCM and SFM dams and foals.. **a.** Observed OTUs, **b.** Simpson, **c.** PD_Whole_Tree **d.** Shannon

Foal samples were grouped into six different age groups determined by the foals’ ages in weeks at the time of sampling. Foals were also grouped by DCM or SFM, gender, access to grazing (access or no access) and where they were housed during the week of sampling (field, stall, or both). Alpha diversity (PD whole tree, Observed OTUs, Shannon, and Simpson) was calculated and compared for all foal and dam groups at each time point [Additional file 5]. There were no differences in alpha diversity (nonparametric t-test, *p* > 0.05) between SFM and DCM when comparing dams, foals by management and time for any of these measures [Additional file 5]. When comparing foals and dams within management types, however, dams had a significantly lower mean diversity than foals (Shannon, Simpson, and PD whole tree, nonparametric t-test, *p* < 0.05 for all measures except PD-whole tree for DCM foals and dams) (Fig. 5). When comparing the six different age groups among foals, week 1 foals had a significantly lower mean diversity than all other weeks (PD whole tree, nonparametric t-test, *p* < 0.01) (Fig. 5c). The core microbiomes of SFM foals and dams were significantly more diverse than DCM dams and foals for all measures (Fig. 6). The average Goods coverage for the dam and foal samples was 98.34%.

**Fig. 7:**
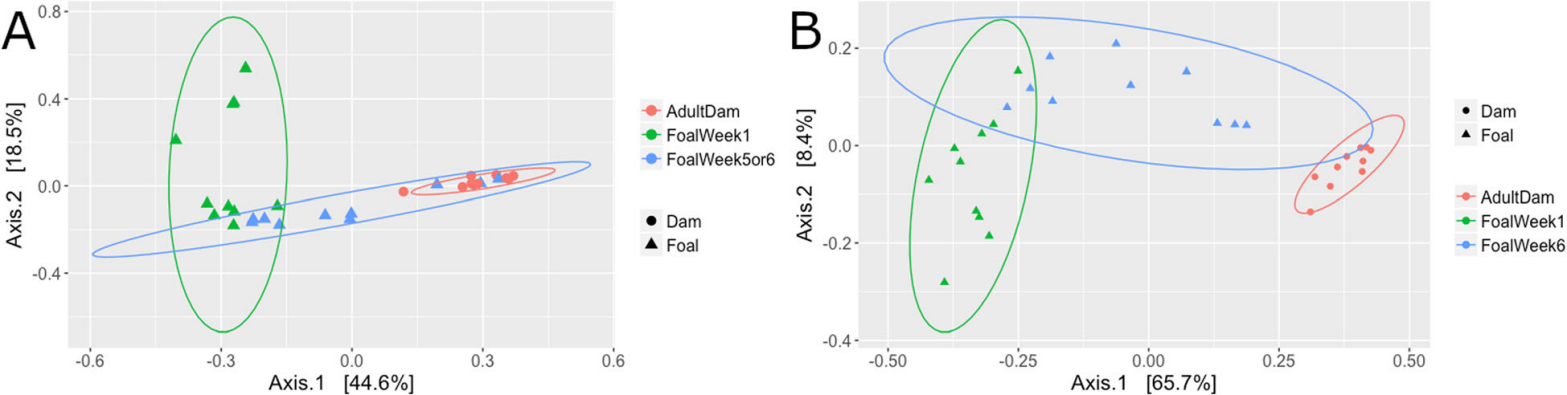
PCoA plot of the relationships between 1-week-old and 5/6-week-old foals as and dams using Bray-Curtis Dissimilarity. Ellipsoids representing a 95% confidence interval were used to surround each dam or foal group. **a.** DCM **b.** SFM

PCoA of Bray-Curtis dissimilarity by management strategy was plotted for 1-week-old foals, 5 or 6-week- old foals, and dams using multidimensional scaling (Fig. 7). As foals age, their microbiomes become more similar to that of their dams, however the domestic dams and their 5/6 weeks old foals (Fig. 7a) clustered more tightly than the semi-feral dams and their 6-week-old foals (Fig. 7b) with higher amount of overlap in the ellipsoids of the domestic dams and their 5/6-week-old foals indicating differences between the two groups in the progress of microbiome development. PCoA plots of weighted and unweighted Unifrac distances demonstrates similar trends for Age and Management [Additional file 6].

Community similarities between and within DCM and SFM foal groups, compared using multivariate ANOSIM and PERMANOVA indicated significant differences between and within DCM and SFM foals based on age, grazing access and housing as well as within each domestication group between age groups [Additional file 7]. These findings show that between study groups, both age and management type affected the foals’ hindgut microbiomes. Significant differences were also found between dams and foals and between SFM and DCM when comparing all dam and foal samples. When analyzing dams only, significant differences were found between SFM and DCM dams indicating that management affects adult horse microbiomes as well as foals.

Pairwise comparisons by age for SFM and DCM foals found significant differences for DCM foals between all ages except for week 2 vs. 3, 3 vs. 4, 3 vs. 5, 3 vs. 6, 4 vs. 5, 4 vs. 6 and 5 vs. 6 foals [Additional file 7]. When comparing all ages in the SFM foals, significant differences were found between all ages except for week 3 vs. 4, 4 vs. 5, 4 vs. 6 and 5 vs. 6 foals. There was more variance between ages in DCM foals, which may indicate that the SFM foals had a more consistent microbiome throughout the study period than DCM foals. Significant differences were found between 6-week-old SFM foals and SFM dams as well as between 6-week-old DCM foals and DCM dams. Therefore, it is clear that these foals’ gut microbiomes had not yet stabilized to that of an adult at 6 weeks of age.

### Differences in community composition

Significantly different OTUs between SFM and DCM foals at different ages as well as SFM and DCM dams are shown on Table 1. The most highly significant (Kruskal-Wallis, *p* < 0.01) taxa belonging to the Firmicutes and Bacteroidetes phyla are shown on Table 2. Genera from the family Lactobacillaceae were found to be significantly more abundant in DCM foals than in SFM foals and semi-feral and domestic dams (Table 2 and Fig. 2c and d). This is interesting because it is a family that contains many lactic acid producing bacteria which have been associated with the onset of starch-induced laminitis [27].

**Table 1:** Guaranteed analysis of DCM dam’s feed (Winbak Original 14 Custom Cube, McCauley Bros., Versailles, KY), which the foal had access to throughout the study period

Crude Protein, min	14.0%
Crude Fat, min	3.5%
Crude Fiber, max	12.0%
Calcium, min	1.0%
Calcium, max	1.5%
Phosphorus, min	0.75%
Copper, min	30 ppm
Selenium, min	0.4 ppm
Zinc, min	100 ppm
Vitamin A, min	4000 IU/lb
Vitamin D, min	800 IU/lb
Vitamin E, min	100 IU/lb

**Table 2:** Highly significantly different groups at the family level between SFM and DCM foals (Kruskal-Wallis, *p* < 0.01). Taxa are shown in the group in which they were enriched

Semi-feral managed foals	Domestic conventionally managed foals
Erysipelotrichaceae gen.	Aerococcaceae gen.
Chlamydiaceae gen.	Lactobacillaceae gen.
Rhodocyclaceae gen.	Porphyromonadaceae gen.
Pasteurellaceae gen.	Corynebacteriaceae gen.
Anaeroplasmataceae gen.	Pseudomonadaceae gen.
S24–7 gen.	Turicibacteraceae gen.
Alcaligenaceae gen.	Sphingomonadaceae gen.
	Clostridiaceae gen.
	Moraxellaceae gen.
	Victivallaceae gen.
	Eubacteriaceae gen.
	Tissierellaceae gen.

Enriched taxa were also analyzed using LEfSe (Linear Discriminant Analysis Effect Size) [28]. DCM and SFM foals were analyzed separately for each of their 6 age groups (Tables 3 and 4). One hundred eighty two taxa were found to be significantly enriched in the different age groups in DCM foals and 151 taxa were found to be significantly enriched in the different ages in SFM foals (*p* < 0.05, Kruskal-Wallis, LDA score > 2.0). Week 5 SFM foals and week 4 DCM foals were found to have *Methanobrevibacter* spp*.* and *Methanobacteriaceae* gen. Enriched in their microbiomes, which are archaea taxa associated with the digestion of complex carbohydrates and methane production. *Fibrobacter* spp*.* and *Fibrobacteraceae* gen. Are also associated with complex plant carbohydrate digestion and were found to be enriched in week 4 SFM foals. *Lactobacillus* spp*.* and *Lactobacillaceae* gen. Were found to be enriched in DCM foals aged 2 and 3 weeks, which reinforces this same finding using a Kruskal-Wallis test stated previously.

**Table 3:** Significantly enriched taxa at the family, genus and species level found in SFM foals from ages 1 to 6 weeks (*p* < 0.05, Kruskal-Wallis, LDA score > 2.0) compared with all other weeks

	Week 1	Week 2	Week 3	Week 4	Week 5	Week 6
**Firmicutes**	Peptostreptococcaceae gen.	*Holdemania spp.*	*Veillonella dispar*		*Coprobacillus spp.*	*Selenomonas noxia*
	*Clostridium spp.*		*Veillonella spp.*			*Mogibacterium spp.*
	*Ruminococcus gnavus*		Christensenellaceae gen.			Mogibacteriaceae gen.
	*Ruminococcus spp.*		Lachnospiraceae gen.			
**Bacteroidetes**		*Odoribacter spp.*		S24_7 gen.		*YRC22 spp.*
		*CF231 spp.*		*Prevotella spp.*		Rikenellaceae gen.
		Paraprevotellaceae gen.		Prevotellaceae gen.		
				*Prevotella copri*		
				Paraprevotellaceae gen.		
**Proteobacteria**	Aeromonadaceae gen.	*Desulfovibrio spp.*			Methylobacteriaceae gen.	*Campylobacter spp.*
					*Helicobacter spp.*	Campylobacteraceae gen.
**Euryarchaeota**			Dehalobacteriaceae gen.		*Methanobrevibacter spp.*	*vadinCA11 spp.*
					Methanobacteriaceae gen.	Methanomassiliicoccaceae gen.
						Methanocorpusculaceae gen.
						*Methanocorpusculum spp.*
**Actinobacteria**			Coriobacteriaceae gen.			
**Fibrobacteres**				*Fibrobacter spp.*		
				Fibrobacteraceae gen.		
				*Fibrobacter succinogenes*		
**Spirochaetes**					*Treponema spp.*	
**Planctomycetes**						Pirellulaceae gen.
**Chlamydiae**						*Chlamydia spp.*
**Verrucomicrobia**						RFP12 gen.

**Table 4:** Significantly enriched taxa at the family, genus and species level found in DCM foals from ages 1 to 6 weeks (*p* < 0.05, Kruskal-Wallis, LDA score > 2.0) compared with all other weeks

	Week 1		Week 2	Week 3	Week 4	Week 5	Week 6
**Firmicutes**	*Butyricicoccus spp.*	*Blautia producta*	*Lactobacillus reuteri*	*Selenomonas ruminantium*	Mogibacteriaceae gen.	Veillonellaceae gen.	*Sarcina spp.*
	*Butyricicoccus pullicaecorum*	*Ruminococcus spp.*	Peptococcaeae gen.	Selenomonas gen.	*Ruminococcus flavefaciens*	*Peptococcus spp.*	*Peptoniphilus spp.*
	*Clostridium perfringens*	*Eubacterium spp.*	*Holdemania spp.*	*Lactobacillus spp.*	*Ruminococcus spp.*	*Coprobacillus spp. 2*	*Rummeliibacillus spp.*
	Clostridium spp.	*Eubacterium dolichum*	*Anaerotruncus spp.*	*Coprococcus spp.*	*RFN20 spp.*	Mogibacteriaceae gen.	*Pseudobutyrivibrio spp.*
	Peptostreptococcaceae gen.	*Oscillospira spp.*	*rc4_4 spp.*	*Clostridium spp.*			*Mogibacterium spp.*
	*Turicibacter spp.*		Peptococcaceae gen.	*Phascolarctobacterium spp.*			Mogibacteriaceae gen.
	Enterococcaceae gen.		*Blautia spp.*	*Roseburia spp.*			Leuconostocaceae gen.
	*Enterococcus spp.*			Lachnospiraceae gen.			*Finegoldia spp.*
	*Enterococcus casseliflavus*			*Lactobacillus spp.*			Tissierellaceae gen.
	*Vagococcus spp.*			Lactobacillaceae gen.			
	*Blautia spp.*			*Dorea spp.*			
**Bacteroidetes**			*Bacteroides ovatus*	*Butyricimonas spp.*	*5_7N15 spp.*	*Bacteroides plebeius*	RF16 gen.
				Odoribacteraceae gen.	Prevotellaceae gen.	*CF231 spp.*	*YRC22 spp.*
				Porphyromonadaceae gen.	*BF311 spp.*	*Prevotella copri*	*Prevotella spp.*
					*Paludibacter spp.*	*Prevotella spp.*	Paraprevotellaceae gen.
					Paraprevotellaceae gen.	Prevotallaceae gen.	
						BS11 gen.	
**Proteobacteria**	Enterobacteriaceae gen.	*Escherichia spp.*	*Actinobacillus spp.*				Oxalobacteraceae gen.
	*Erwinia dispersa*	*Escherichia coli*	Pasteurellaceae gen.				
	*Erwinia spp.*	*Sphingomonas spp.*					
	*Citrobacter spp.*	*Morganella spp.*					
	*Proteus spp.*	*Klebsiella spp.*					
**Actinobacteria**	*Eggerthella spp.*				*Actinomyces spp. 2*	Actinomycetaceae gen.	*Corynebacterium spp.*
	*Eggerthella lenta*						Corynebacteriaceae gen.
**Verrucomicrobia**				*Akkermansia muciniphila*	*Akkermansia spp.*		R4_41B gen.
							RFP12 gen.
**Euryarchaeota**					Dehalobacteriaceae gen.		*Methanimicrococcus spp.*
					*Methanobrevibacter spp.*		Methanosarcinaceae gen.
					Methanobacteriaceae gen.		Methanomassiliicoccaceae gen.
							*vadinCA11 spp.*
							Methanocorpusculaceae gen.
							*Methanocorpusculum spp.*
**Spirochaetes**					*Treponema spp.*		Spirochaetaceae gen.
**Chlamydiae**							*Chlamydia spp.*
**Planctomycetes**							Pirellulaceae gen.
**Synergistetes**							Synergistaceae gen.
**Cyanobacteria**							Synechococcaceae gen.
							*Synechococcus spp.*

### Predicted functional analysis of foal and dam hindgut microbiome

Functional potential of communities was inferred using PICRUSt [29] to generate predictions based on Kyoto Encyclopedia of Genes and Genomes (KEGG) pathways at Level 3 [30]. These predictions were then sorted into 6 different digestion related categories [Additional file 8].

**Fig. 8:**
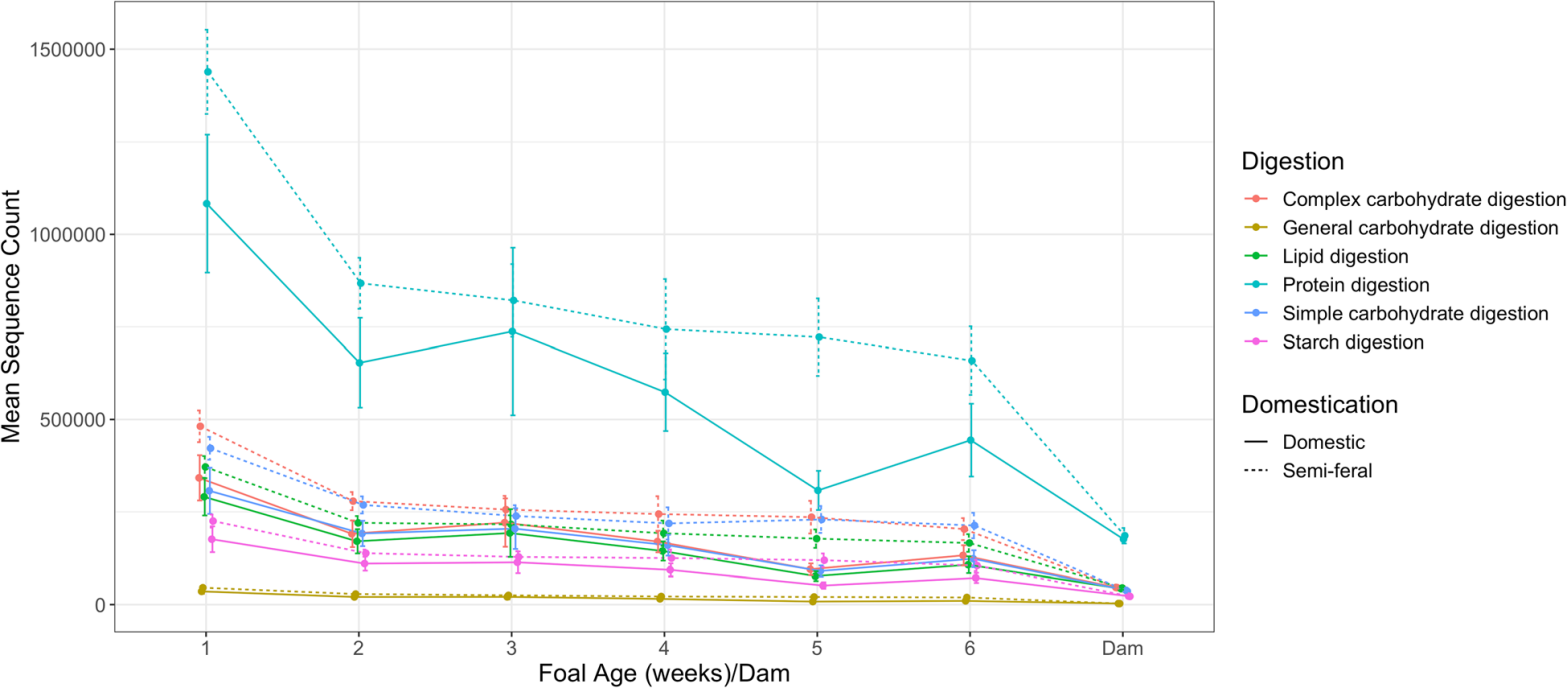
Mean sequence counts of the taxa responsible for the major digestion functions of semi-feral and domestic foals from week 1 to week 6 of life and semi- feral and domestic dams. Standard error indicated

Significant differences were found between the 6 different age groups and DCM and SFM for all types of digestion: general carbohydrate, lipid, protein, complex carbohydrate, starch and simple carbohydrate (*p* < 0.05, Kruskal-Wallis). Week 1 DCM and SFM foals had the greatest amount of general carbohydrate-, lipid-, protein-, complex carbohydrate-, starch- and simple carbohydrate-digesting bacteria when compared to the rest of the age groups, including dams. This finding is most likely due to nutrient-rich colostrum and mare’s milk during the foal’s first week of life and the gradual decrease in nutrient content as time progressed. As the foals aged, it was apparent that the abundance of the OTUs contributing to each digestion type gradually decreased to reach levels similar to those of their dams (Fig. 8). Both SFM and DCM foals at every age group were found to have significantly higher levels in all types of digestion than SFM and DCM dams (*p* < 0.05, Kruskal-Wallis). Significant differences were also found between SFM and DCM foals with SFM foals having a significantly higher mean sequence count in the OTUs contributing to each type of digestion (*p* < 0.001, Kruskal-Wallis). No significant differences were found in the digestion types between SFM and DCM dams, which may indicate that SFM and DCM adult microbiomes are functionally similar.

## Discussion

We report significant effects of management type and age on the hindgut microbiome in foals and dams, identifying differences in the microbiome structure of semi-feral and domestically managed horses. Bacteroidetes and Firmicutes were the dominant phyla in all samples as has been reported elsewhere [31, 32]. SFM foals differed from DCM foals in enrichment of specific Firmicutes families (Erysipelotrichaceae and Streptococcaceae), while DCM foals were enriched in Lactobacillaceae and Christiansellaceae. For Bacteroidetes, SFM foals were enriched in Bacteroidaceae, while DCM foal samples contained more Paraprevotellaceae. Profiles generated for DCM foals mirrored other studies of foal microbiome development [32]. Differences were found in abundances of specific OTUs between SFM and DCM foals as well as in their hindgut microbial communities as a whole. Our data suggests that DCM foals possess a microbiome more similar to that of an adult at an earlier age than SFM foals. This could be due to the DCM dams and foals having more limited and uniform diets than SFM foals and dams. The accessibility of a variety of plant materials as well as exposure to numerous other horses in social groups likely provided a more varied exposure to the SFM foals from the beginning of life, and suggests that dietary and environmental features could shape the gut microbiome in foals from a young age. The expanded membership and distribution of taxa found in the core microbiomes of SFM dams and foals points to differences in community structure based on management that could confer greater redundancy, and thus enhanced resilience to dietary change and/or stress.

To determine the stabilization period of the SFM and DCM foal microbiomes, it would be necessary to follow these subjects for a longer period of time. In previous studies, researchers found that domestic conventionally managed foals had a stable, adult-like microbiome at 1 to 2 months old [18, 19]. In the current study, the gut microbiomes of both SFM and DCM foals remained significantly different than their dams at 5 or 6 weeks of life [Additional file 7]. Week 5 and 6 DCM foals and week 6 SFM foals were found to have significantly higher levels in all types of digestive functions inferred by PICRUSt analysis than their dams (Fig. 8). Therefore, these foals did not have an adult-like microbiome with respect to composition and function during this study period but may have established a stable one in the subsequent weeks after sampling had ended.

PICRUSt analysis [29] to infer the digestion functionality of the foals’ microbiomes suggested that week 1 foals had the greatest amount of general carbohydrate-, lipid-, protein-, complex carbohydrate-, starch- and simple carbohydrate-digesting capability. The most abundant type of digestion in foals was inferred to be protein digestion followed by complex carbohydrate, simple carbohydrate, lipid, starch and general carbohydrate digestion. Levels of each type of digestion gradually decreased as the foal aged, but was inferred to be higher than their dams throughout the study. Mare milk in the first week of lactation has been estimated to contain 2.64% protein, 2.07% fat, 6.15% lactose, 23.16% milk urea nitrogen and a somatic cell count of 40,640 cells/mL [33]. Both fat content and protein decrease in mare’s milk as the lactation weeks progressed, which may explain why inferences of bacterial counts associated with both protein and lipid digestion were found to have decreased as the foals aged in the current study.

Despite the relatively small number of foals and dams in this study (n_foals_ = 20, n_dams_ = 20), there were clear differences between SFM and DCM groups. Management factors such as diet were likely a major driver of microbiome structure. This is because DCM foals had access to their dam’s concentrate feed as well as hay and limited forage while SFM foals only had access to natural forage. Differences between DCM and SFM foals’ microbiomes over time could be due to the changing diet of the DCM group throughout the study period; from no grazing in week 1 to limited access for the remaining weeks as well as increasing access to the dams’ grain. These changes in diet may also contribute to the differences found between ages in DCM foals. In addition to diet, other management factors that could have been driving differences between SFM and DCM horses in this study included housing, differential contact with other horses, and differences in the frequency of human handling.

The differences found in this study between SFM and DCM horses were shown to be related to management and not breed, in agreement with previous reports [24], however there were significant differences due to study between the current study and the EMP data [Additional files 3 and 4]. PCoA plots of Bray-Curtis distances show almost complete overlap between the pony and the Standardbred samples (Fig. 3a), while there were two distinct groups of samples based on management (Fig. 3b). There was also clustering due to study (Fig. 3c), which points to differences due to sample handling between the EMP (Equine Microbiome Project) protocols and the current study. Unfortunately there were no SFM Standardbred horses to include in this study to make this comparison.

This study provides insight into how management affects the structure, function, and development of the equine microbiome starting at birth. Since SFM and DCM dams also had distinct microbiomes from one another, it is apparent that management factors such as diet, socialization and housing impact horses in their adult life as well. Further study is needed to determine the relative importance of specific management factors in shaping the microbiomes of horses. In this study SFM foals and dams had a higher amount of social interaction with other horses and more variable grazing access than DCM foals and dams as well as greater variability in climate, environmental exposure to pathogens and stress levels. DCM foals had a greater level of human contact and less variable diets and environmental conditions due to housing and handling. Horses are adapted to be continuous grazers, which can be difficult to achieve in the domestic setting. Domestic horse diets are higher in starch and other easily fermented sugars, leading to higher prevalence of diseases like starch-induced laminitis and gastric ulcers [23]. Management strategies more closely resembling SFM may modulate the microbiome toward a healthier balance and reduce the incidence of diet related illnesses.

## Conclusions

This study demonstrates that management impacts the structure and inferred function of the foal hindgut microbiome during development in the first 6 weeks of life as well as for adult horses. The enhanced diversity of key groups (Verrucomicrobia (RFP12), Ruminococcaceae, *Fusobacterium* spp., and *Bacteroides* spp.), higher number of taxa and OTUs found in SFM dams and foals, and expanded inferred functional repertoire suggest greater functional redundancy, stability, and digestive capacity for the gut microbiomes of SFM horses. Greater abundance of lactic acid bacteria in DCM dams and foals indicates early community adaptation to concentrate feeds. Further research is needed to identify specific management factors that are most significant for gut microbiome health and function in horses, and how the management of domestic horses may be informed by knowledge of semi-feral horses in a more natural state.

## Methods

### Subjects

Ten DCM Standardbreds and ten SFM Shetland-type pony foals and dams were included in this study. There were seven males and three females in the SFM group of foals and five males and five females in the DCM group of foals. All foals and dams included in this study were healthy at birth with no serious gastrointestinal problems and no administration of antimicrobials, anti-inflammatories or supplemental products such as probiotics or digestion supplements at any stage during sampling.

DCM dams were Standardbred broodmares maintained by Winbak Farm, Chesapeake City, Maryland. Each DCM foal was born and kept with their dam in a stall during their first week of life. The DCM foals and dams then made the transition to a small paddock for approximately 8 h per day until they reached 45 days of age. In most instances, there were two foal-dam pairs per paddock. During the rest of the day, each foal-dam pair was enclosed in a stall with free access to hay. After their first 45 days of life, the DCM foals and dams were permanently located in a large pasture with other foal-dam pairs. DCM foals had access to their dam’s feed (Table 1) throughout the study period and had access to grass at the beginning of their second week of life.

The Shetland-type pony foals were born into a semi-feral herd maintained since 1994 at the University of Pennsylvania School of Veterinary Medicine in Kennett Square, Pennsylvania. DNA-based parentage is confirmed for all offspring (Gluck Equine Parentage Testing Laboratory, University of Kentucky, Lexington, KY). At the time of this study, the herd consisted of 11 harem groups and one bachelor band with a total of 105 animals. The ponies had no history of laminitis or major gastrointestinal diseases. Handling by humans in the semi-feral herd was limited to required preventative health care (daily observation, annual vaccinations and deworming when necessary) completed by highly skilled technicians experienced with these procedures using positive reinforcement. In addition, each SFM foal received a 30-min “gentling” experience of positive reinforcement-based acclimation to human interaction with 21 specific compliance goals including touch all over the body, simulated veterinary examination and routine health care procedures, introduction of a halter, and introduction to leading if time allowed when they were between the age of 2 and 4 weeks old. The environment of the semi-feral herd consisted of a 40-acre enclosure with natural forages and water sources as well as natural shelters such as hedges and light forest.

### Sampling protocol

Foals were sampled on a once weekly schedule from birth. Rectal swab samples were taken from foals once a week until the foal was either 5 or 6 weeks old. All ten SFM foals were sampled weekly through week 6. Six DCM foals were sampled through week 6 and the remaining 4 foals were sampled through week 5 due to the inability to access them for sampling during their sixth week of life. Each dam was sampled once at week 5 or 6 post-partum during the study period. Swab samples were collected in triplicate using sterile cotton-tipped swabs, stored on ice for no more than an hour, then placed in a bead tube containing 750 μl of bead solution (MO BIO Laboratories Inc., Carlsbad, CA). The tubes were then stored in a freezer at -20 °C until extraction.

### DNA extraction and sequencing

Genomic DNA was extracted from each swab sample using MO BIO Laboratories PowerFecal DNA Isolation Kit® (MO BIO Laboratories Inc., Carlsbad, CA) as directed except 50 μL of solution C6 was used during the last step instead of 100 μL and this solution was left to sit for 5 min in the spin filter before the final centrifugation to maximize yield. Total DNA concentration in each sample was determined using a Qubit® (ThermoFisher Scientific, Waltham, MA) fluorometer and sample quality was determined using a Nanodrop® (ThermoFisher Scientific, Waltham, MA) spectrophotometer.

One sample from each triplicate set with the highest DNA concentration and best absorbance ratio (260/280 = 1.8) was sequenced. Triplicate sample sets with low DNA quantity and quality were concentrated and cleaned by ethanol precipitation. The V4-V5 variable region of the 16S rRNA gene was amplified using universal primers (515yF 3′-GTGYCAGCMGCCGCGGTAA-5′/926pfR 3′-CCGYCAATTYMTTTRAGTTT-5′) and sequenced using normalized DNA pools and dual-barcoded Illumina MiSeq library preparation (RTL Genomics, Lubbock, TX). Primer choice was based on established Earth Microbiome Protocols [34]. A sequencing blank was prepared using all steps in the DNA extraction protocol for the PowerFecal DNA Isolation Kit® (MO BIO Laboratories Inc., Carlsbad, CA) with water, and sequenced as described above.

### Bioinformatics analysis

QIIME (Quantitative Insights Into Microbial Ecology) version 1.9.1 was used for microbial data processing and statistical analysis [35]. FLASh (Fast Length Adjustment of SHort reads) was used with default parameters to merge paired-end reads [36]. Approximately 5% of single reads failed to pair and were removed. FastQC was used to validate read quality and consistency [37]. In QIIME version 1.9.1, sequence reads were filtered for length (400 bp), assessed for quality (Phred score of 20, and 3 maximum consecutive low-quality base calls), and primers were removed using the split_seqs_fastq command. Reads were rarefied to the lowest sequence count of 3500 reads per sample for beta diversity analysis.

Open reference OTUs (closed reference clustering followed by a de novo step) were picked with UCLUST [38] at 97% identity against the Greengenes version 13_8 database [38, 39]. OTUs observed only once or twice were filtered out of the OTU table. Sequence counts were subtracted for OTUs found in 100% of the samples in common with the sequencing blank, and the OTU table was normalized using cumulative sum scaling (CSS). Alpha diversity (PD whole tree, Observed OTUs, Shannon, and Simpson) was calculated using the normalized OTU table and the [rep_set.tre] output from the OTU picking step. These measures were compared between groups and time points using pair-wise t-tests. Beta diversity was calculated (weighted and unweighted Unifrac and Bray-Curtis distances) and compared using ANOSIM and PERMANOVA to determine differences over time. Differences in OTU abundance (group significance) were tested using Kruskal-Wallis in QIIME version 1.9.1 [35]. Differences in taxa abundance were identified using pair-wise t-tests. The core microbiomes of SFM and DCM foals and dams (taxa present in 95% of samples in each group) were identified in QIIME version1.9.1 using the [core_microbiome.py] script [35].

Enriched taxa by management group and time were identified using LEfSe [28]. Statistical analysis and visualization were completed using R [39, 40]. PICRUSt [29] was used on the Galaxy instance (http://huttenhower.sph.harvard.edu/galaxy/) to infer functional potential of each sample’s gut bacterial community using closed reference OTUs generated against the Greengenes version 13_5 database [38, 39]. Briefly, OTUs were normalized by copy number, metagenome predictions were made and categorized to identify enriched KEGG functions [30].

### Effect of breed on Horse’s hindgut microbiome

Comparison of breed and management effects on the gut microbiome was conducted in order to justify the use of Standardbred foals and dams to Shetland-type pony foals and dams in this study. Data from Shetland-type ponies and Standardbred comparators were selected from the EMP database [26], a collection of 16S surveys and metadata from fecal samples of 285 horses to date. EMP data selected for this study included sequencing data from healthy individuals that had not received deworming medication or antibiotics within 30 days of sampling. Eight adult ponies and nineteen Standardbred adult horses were compared with the dams in this study. All of the EMP horses and ponies were under DCM management.

The EMP fecal samples were collected and processed using standard protocols that only differed from the current study in sampling methodology. Briefly, freshly voided fecal samples were collected in 20% DNA Shield (Zymo, Irvine, CA) and stored at 4 °C prior to shipping to the lab. Once received, DNA was extracted using the same methodology and sequenced using the Illumina MiSeq platform and primers as described above for the dam samples. Raw sequence data from the EMP horses was combined with the dam data from the present study prior to the OTU picking step in QIIME version 1.9.1. Subsequent bioinformatic analysis was done as described above.

## Supplementary Information

**Additional file 1.** Sequence counts of blank sequencing control. Absolute abundance counts for blank sequencing control. Highlighted in blue are OTUs found in 100% of the experimental samples.

**Additional file 2.** Comparison of EMP horses and Current study. Phyla level comparison of Pony and Standardbred fecal 16S rRNA profiles from the current study (adult SFM and DCM horses) and the EMP Database.

**Additional file 3.** Group significance based on breed and study. Group significance test for breed by Kruskal-Wallis (GS_KW_SBP) and nonparametric t-test (GS_NPT_SBP). Group significance test for study by Kruskal-Wallis (GS_KW_Study)

**Additional file 4.** Alpha diversity boxplots comparing EMP horses with the current study by breed. Boxplots of alpha diversity measures: Observed OTUs (A), Simpson (B), PD_Whole_tree (C), and Shannon (C) comparing EMP horses and the current study by breed.

**Additional file 5.** Alpha diversity measures for Dams and Foals. Calculated alpha diversity measures for all Dam and Foal samples from the CSS normalized OTU table and results of t-tests for each alpha diversity comparing foals by week and dams.

**Additional file 6.** PCoA plots of Unifrac distances. PCoA plots of unweighted Unifrac distances by Management (A) and Age (B). PCoA plots of weighted Unifrac distances colored by Management (C) and Age (D).

**Additional file 7.** Significant ANOSIM and PERMANOVA comparisons. Statistical analysis of different foal and dam groups using ANOSIM and PERMANOVA tests. (note that foal gender was not found to be a significant factor).

**Additional file 8.** KEGG Functional categories. KEGG functions categorized into six different types of digestion.

## Data Availability

The datasets generated during this study have been deposited in the NCBI Sequence Read Archive: https://www.ncbi.nlm.nih.gov/sra, Bioproject: PRJNA647744, Accession numbers: SAMN15597322- SAMN15597482.

## Abbreviations

DCM: domestic conventionally managed
SFM: semi-feral managed
OTU: operational taxonomic unit
PICRUSt: Phylogenetic Investigation of Communities by Reconstruction of Unobserved States
PCoA: Principle Coordinate Analysis
LEfSe: Linear Discriminant Analysis Effect Size
EMP: Equine Microbiome Project
QIIME: Quantitative Insights Into Microbial Ecology
FLASh: Fast Length Adjustment of SHort reads
CSS: Cumulative Sum Scaling
KEGG: Kyoto Encyclopedia of Genes and Genomes
