# Supplementary material for: Development of the equine hindgut microbiome in semi-feral and domestic conventionally-managed foals
Source: Anim Microbiome. 2020 Nov 23;2:43. doi: 10.1186/s42523-020-00060-6 (PMC7807438; doi:10.1186/s42523-020-00060-6)
Supplement: Supplementary file 2 — Additional file 2. Comparison of EMP horses and Current study. Phyla level comparison of Pony and Standardbred fecal 16S rRNA profiles from the current study (adult SFM and DCM horses) and the EMP Database. [file 42523_2020_60_MOESM2_ESM.docx]

Additional file 1

Comparison of EMP horses and Current study


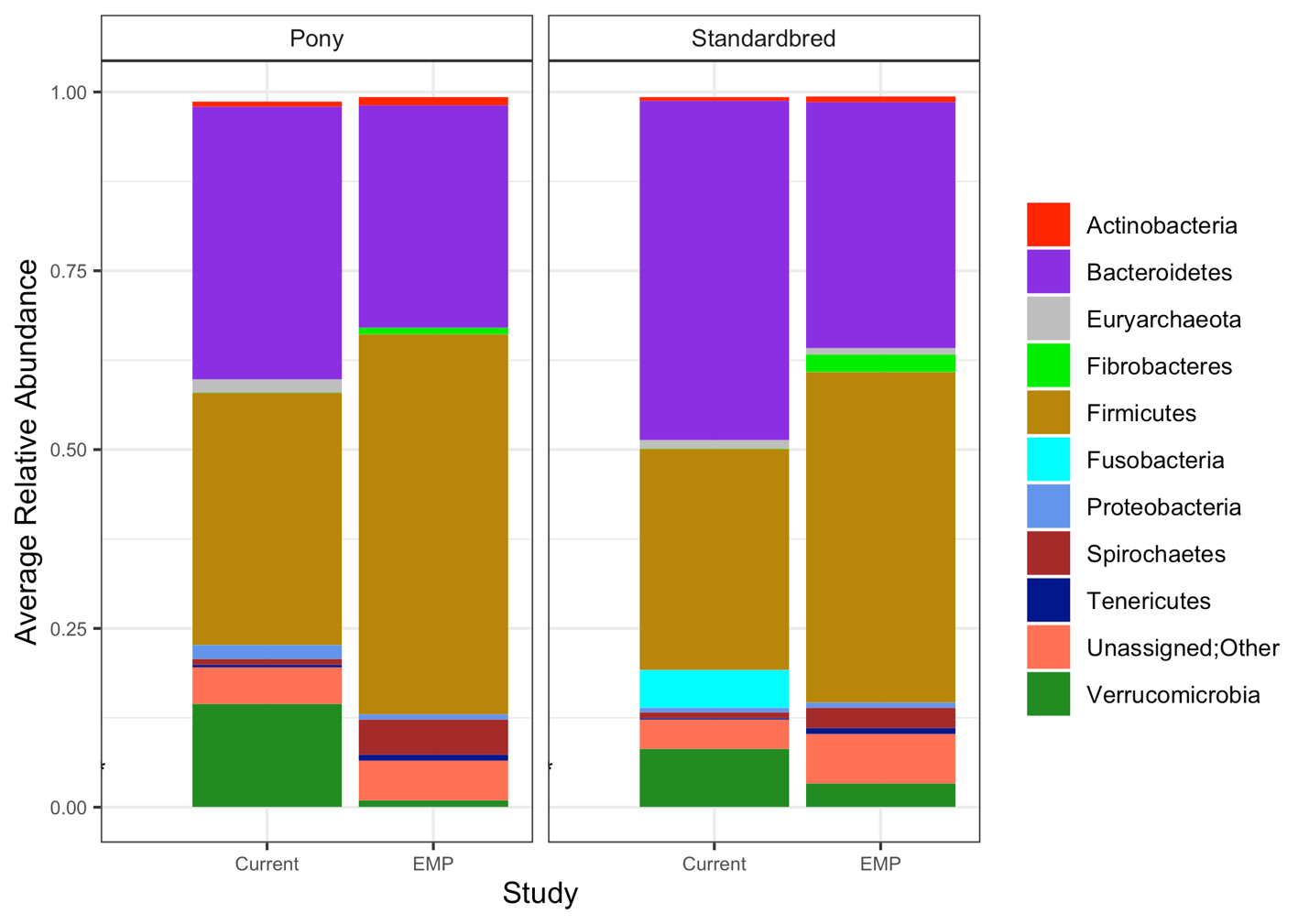


Phyla level comparison of Pony and Standardbred fecal 16S rRNA profiles from the current study (adult SFM and DCM horses) and the Equine Microbiome Project Database.
