# Supplementary figures and images for: Development of the equine hindgut microbiome in semi-feral and domestic conventionally-managed foals
Source: Anim Microbiome. 2020 Nov 23;2:43. doi: 10.1186/s42523-020-00060-6 (PMC7807438; doi:10.1186/s42523-020-00060-6)

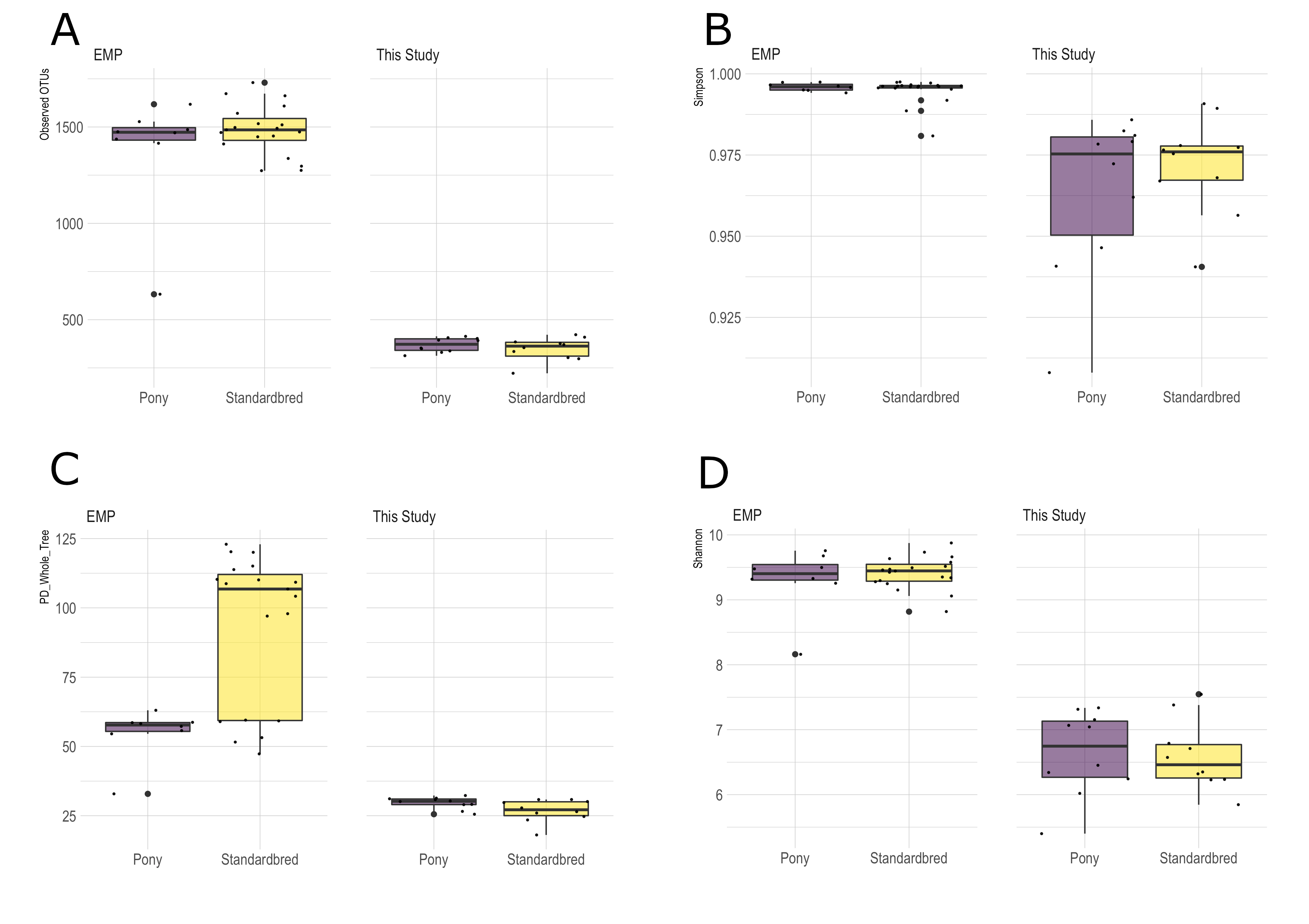

Supplement: Supplementary file 4 — Additional file 4. Alpha diversity boxplots comparing EMP horses with the current study by breed. Boxplots of alpha diversity measures: Observed OTUs (A), Simpson (B), PD_Whole_tree (C), and Shannon (C) comparing EMP horses and the current study by breed. [file 42523_2020_60_MOESM4_ESM.tiff]

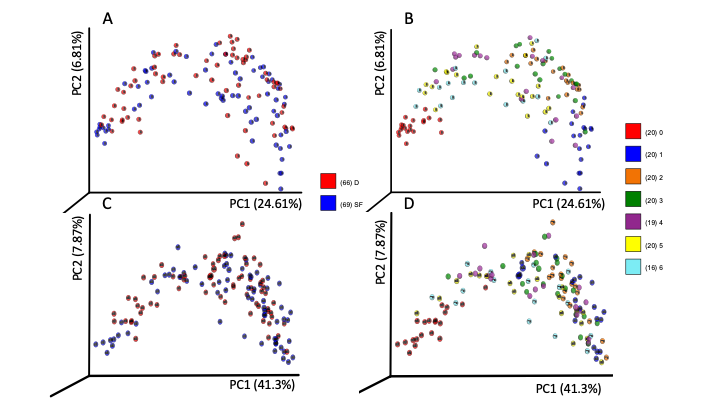

Supplement: Supplementary file 6 — Additional file 6. PCoA plots of Unifrac distances. PCoA plots of unweighted Unifrac distances by Management (A) and Age (B). PCoA plots of weighted Unifrac distances colored by Management (C) and Age (D). [file 42523_2020_60_MOESM6_ESM.tiff]
