# Supplementary material for: Development of the equine hindgut microbiome in semi-feral and domestic conventionally-managed foals
Source: Anim Microbiome. 2020 Nov 23;2:43. doi: 10.1186/s42523-020-00060-6 (PMC7807438; doi:10.1186/s42523-020-00060-6)
Supplement: Supplementary file 5 — Additional file 5. Alpha diversity measures for Dams and Foals. Calculated alpha diversity measures for all Dam and Foal samples from the CSS normalized OTU table and results of t-tests for each alpha diversity comparing foals by week and dams. [file 42523_2020_60_MOESM5_ESM.docx]

Additional file 5

Calculated alpha diversity measures for all Dam and Foal samples from the CSS normalized OTU table. Management (Man): SFM = Semi-feral, DCM = Domestic.

| **Sample** | **PD_whole_tree** | **shannon** | **simpson** | **observed_otus** | **man** | **Age** | **Foal_Dam** |
| --- | --- | --- | --- | --- | --- | --- | --- |
| D1.1A | 48.36596 | 8.593871982 | 0.997065798 | 454 | SFM | 0 | Dam |
| D10.1A | 45.53785 | 8.373097163 | 0.996533048 | 395 | SFM | 0 | Dam |
| D12.1A | 51.34573 | 8.481646311 | 0.996782579 | 432 | DCM | 0 | Dam |
| D13.1A | 48.16361 | 8.430091455 | 0.996605298 | 427 | DCM | 0 | Dam |
| D14.1A | 31.32215 | 7.772635178 | 0.994921345 | 255 | DCM | 0 | Dam |
| D15.1A | 54.17404 | 8.618605199 | 0.997055735 | 478 | DCM | 0 | Dam |
| D18.1A | 52.81693 | 8.573242077 | 0.996959554 | 440 | DCM | 0 | Dam |
| D2.1C | 42.14032 | 8.169975334 | 0.996029054 | 342 | SFM | 0 | Dam |
| D20.1A | 49.65654 | 8.519596304 | 0.996909799 | 428 | DCM | 0 | Dam |
| D21.1A | 50.19443 | 8.568473106 | 0.997035034 | 442 | DCM | 0 | Dam |
| D25.1B | 46.63016 | 8.313742179 | 0.996313502 | 391 | DCM | 0 | Dam |
| D26.1C | 52.67686 | 8.679648226 | 0.997212611 | 485 | DCM | 0 | Dam |
| D28.1B | 38.18892 | 8.037257198 | 0.995672031 | 313 | DCM | 0 | Dam |
| D3.1C | 45.48569 | 8.462186892 | 0.996796275 | 412 | SFM | 0 | Dam |
| D4.1A | 44.45919 | 8.374977462 | 0.996509098 | 403 | SFM | 0 | Dam |
| D5.1C | 49.86164 | 8.548854099 | 0.996961343 | 443 | SFM | 0 | Dam |
| D6.1A | 47.59727 | 8.584715103 | 0.997100906 | 445 | SFM | 0 | Dam |
| D7.1B | 46.42556 | 8.571673039 | 0.997060046 | 443 | SFM | 0 | Dam |
| D8.1A | 45.11077 | 8.355870844 | 0.99652426 | 391 | SFM | 0 | Dam |
| D9.1A | 47.7953 | 8.53107474 | 0.996954123 | 433 | SFM | 0 | Dam |
| F1.1A | 49.35495 | 8.983520251 | 0.997627988 | 626 | SFM | 1 | Foal |
| F1.2C | 54.52792 | 8.778947407 | 0.997270398 | 555 | SFM | 2 | Foal |
| F1.3B | 46.00764 | 8.715121003 | 0.997197685 | 515 | SFM | 3 | Foal |
| F1.4A | 68.50295 | 9.1046911 | 0.997794318 | 700 | SFM | 4 | Foal |
| F1.5A | 66.66963 | 9.144178896 | 0.997907033 | 704 | SFM | 5 | Foal |
| F1.6A | 78.577 | 9.430664657 | 0.998204588 | 876 | SFM | 6 | Foal |
| F10.1C | 25.93197 | 7.932105004 | 0.995322316 | 293 | SFM | 1 | Foal |
| F10.2B | 47.4502 | 8.972460967 | 0.997647002 | 619 | SFM | 2 | Foal |
| F10.3C | 63.34598 | 9.239848293 | 0.998016961 | 757 | SFM | 3 | Foal |
| F10.4A | 69.89832 | 9.298118248 | 0.998083036 | 789 | SFM | 4 | Foal |
| F10.5B | 71.95714 | 9.342416449 | 0.998168158 | 801 | SFM | 5 | Foal |
| F10.6B | 39.70962 | 8.278292939 | 0.99631657 | 359 | SFM | 6 | Foal |
| F12.1A | 40.99516 | 8.656185892 | 0.997102888 | 503 | DCM | 1 | Foal |
| F12.2A | 41.49896 | 8.618056522 | 0.996974061 | 488 | DCM | 2 | Foal |
| F12.3A | 58.4254 | 9.21460724 | 0.997978632 | 749 | DCM | 3 | Foal |
| F12.4A | 61.41808 | 9.179818938 | 0.997948056 | 722 | DCM | 4 | Foal |
| F12.5A | 65.30295 | 9.086240654 | 0.997842955 | 653 | DCM | 5 | Foal |
| F13.1A | 42.40794 | 8.619381297 | 0.996997854 | 495 | DCM | 1 | Foal |
| F13.2A | 35.04888 | 8.115583825 | 0.995690782 | 347 | DCM | 2 | Foal |
| F13.3A | 53.32614 | 8.875639254 | 0.997485098 | 586 | DCM | 3 | Foal |
| F13.4A | 64.11215 | 9.073785549 | 0.997811795 | 660 | DCM | 4 | Foal |
| F13.5A | 49.1241 | 8.610127315 | 0.997070769 | 473 | DCM | 5 | Foal |
| F13.6A | 61.04622 | 8.902981013 | 0.997533585 | 599 | DCM | 6 | Foal |
| F14.1B | 39.96383 | 8.676019691 | 0.997189946 | 487 | DCM | 1 | Foal |
| F14.2A | 50.93003 | 8.788202302 | 0.997295684 | 560 | DCM | 2 | Foal |
| F14.3A | 60.59065 | 8.912830491 | 0.997540276 | 609 | DCM | 3 | Foal |
| F14.4A | 56.80755 | 9.015832524 | 0.997765249 | 626 | DCM | 4 | Foal |
| F14.5C | 51.48762 | 8.655990004 | 0.997116591 | 489 | DCM | 5 | Foal |
| F14.6A | 45.66077 | 8.443570393 | 0.996739371 | 417 | DCM | 6 | Foal |
| F15.1A | 44.65324 | 8.84914565 | 0.997439723 | 574 | DCM | 1 | Foal |
| F15.2A | 47.85385 | 9.047564434 | 0.997781102 | 636 | DCM | 2 | Foal |
| F15.3B | 39.76947 | 8.559158476 | 0.996880217 | 467 | DCM | 3 | Foal |
| F15.4A | 61.41788 | 9.058324848 | 0.997732898 | 681 | DCM | 4 | Foal |
| F15.5C | 32.50743 | 7.707792273 | 0.994422177 | 244 | DCM | 5 | Foal |
| F18.1A | 15.78619 | 7.209292542 | 0.992518912 | 163 | DCM | 1 | Foal |
| F18.2A | 35.31565 | 8.33028318 | 0.996316056 | 403 | DCM | 2 | Foal |
| F18.3A | 47.1377 | 8.793792804 | 0.997359215 | 538 | DCM | 3 | Foal |
| F18.4A | 45.04016 | 8.638333884 | 0.997079105 | 479 | DCM | 4 | Foal |
| F18.5C | 57.82227 | 8.907065591 | 0.997469943 | 600 | DCM | 5 | Foal |
| F2.1A | 32.77209 | 8.519567082 | 0.996787047 | 452 | SFM | 1 | Foal |
| F2.2A | 62.76936 | 9.236162818 | 0.997994343 | 755 | SFM | 2 | Foal |
| F2.3A | 70.93902 | 9.296680321 | 0.998061884 | 802 | SFM | 3 | Foal |
| F2.4A | 38.44794 | 8.37817319 | 0.996371817 | 431 | SFM | 4 | Foal |
| F2.5A | 45.20357 | 8.621494376 | 0.996992186 | 491 | SFM | 5 | Foal |
| F2.6B | 51.28476 | 8.77467869 | 0.997293562 | 544 | SFM | 6 | Foal |
| F20.1A | 26.30883 | 7.750370608 | 0.994657506 | 251 | DCM | 1 | Foal |
| F20.2A | 50.64566 | 8.811685124 | 0.997378748 | 547 | DCM | 2 | Foal |
| F20.3A | 69.50665 | 9.237119177 | 0.997969815 | 766 | DCM | 3 | Foal |
| F20.4C | 36.84064 | 8.234645663 | 0.996132399 | 372 | DCM | 4 | Foal |
| F20.5B | 52.34996 | 8.754238334 | 0.997215625 | 546 | DCM | 5 | Foal |
| F3.1B | 26.49531 | 8.010766306 | 0.995541194 | 309 | SFM | 1 | Foal |
| F3.2A | 51.87147 | 8.832901895 | 0.997429385 | 555 | SFM | 2 | Foal |
| F3.3A | 51.49705 | 8.942858227 | 0.997540532 | 611 | SFM | 3 | Foal |
| F3.4A | 49.40872 | 8.528764296 | 0.996554028 | 489 | SFM | 4 | Foal |
| F3.5C | 69.4478 | 9.152359401 | 0.997869636 | 714 | SFM | 5 | Foal |
| F3.6C | 69.95336 | 9.198955359 | 0.997965157 | 729 | SFM | 6 | Foal |
| F4.1A | 35.80092 | 8.292514036 | 0.996223501 | 397 | SFM | 1 | Foal |
| F4.2A | 55.74178 | 9.17239815 | 0.997943305 | 702 | SFM | 2 | Foal |
| F4.3A | 67.5297 | 9.236481453 | 0.997961562 | 772 | SFM | 3 | Foal |
| F4.4A | 58.70391 | 9.133291181 | 0.997851709 | 709 | SFM | 4 | Foal |
| F4.5A | 54.16708 | 8.81365141 | 0.997309024 | 569 | SFM | 5 | Foal |
| F4.6C | 53.95298 | 8.825502615 | 0.997366724 | 564 | SFM | 6 | Foal |
| F5.1A | 35.27547 | 8.473167927 | 0.996717886 | 429 | SFM | 1 | Foal |
| F5.2A | 47.22598 | 8.884605248 | 0.997525877 | 564 | SFM | 2 | Foal |
| F5.3A | 51.00094 | 8.646205755 | 0.997003087 | 503 | SFM | 3 | Foal |
| F5.4A | 55.80008 | 8.993107708 | 0.99767375 | 636 | SFM | 4 | Foal |
| F5.5A | 50.40579 | 8.740142444 | 0.997214382 | 536 | SFM | 5 | Foal |
| F5.6A | 60.52622 | 9.120346136 | 0.997887389 | 675 | SFM | 6 | Foal |
| F6.1A | 29.74607 | 8.123207108 | 0.995863708 | 342 | SFM | 1 | Foal |
| F6.2B | 49.04286 | 9.010220231 | 0.997711953 | 638 | SFM | 2 | Foal |
| F6.3C | 50.80564 | 8.89136303 | 0.997458797 | 606 | SFM | 3 | Foal |
| F6.4A | 41.6316 | 8.353875712 | 0.996650065 | 358 | SFM | 4 | Foal |
| F6.5A | 56.61129 | 8.825375583 | 0.997329602 | 566 | SFM | 5 | Foal |
| F6.6C | 59.77721 | 8.920431806 | 0.997500031 | 613 | SFM | 6 | Foal |
| F7.1A | 27.5361 | 7.874163242 | 0.995059161 | 290 | SFM | 1 | Foal |
| F7.2C | 41.29239 | 8.607112465 | 0.996996212 | 487 | SFM | 2 | Foal |
| F7.3A | 49.32106 | 8.877817605 | 0.997496911 | 577 | SFM | 3 | Foal |
| F7.4A | 37.05295 | 8.473146872 | 0.996673284 | 444 | SFM | 4 | Foal |
| F7.5B | 58.96784 | 8.951559139 | 0.997589797 | 609 | SFM | 5 | Foal |
| F7.6A | 69.59301 | 9.271313097 | 0.998083601 | 751 | SFM | 6 | Foal |
| F8.1C | 36.6486 | 8.673469519 | 0.997123165 | 502 | SFM | 1 | Foal |
| F8.2C | 43.52888 | 8.734022956 | 0.99723209 | 524 | SFM | 2 | Foal |
| F8.3C | 39.69781 | 8.509950014 | 0.996765736 | 455 | SFM | 3 | Foal |
| F8.4B | 34.02907 | 8.105290755 | 0.995830584 | 315 | SFM | 4 | Foal |
| F8.5A | 59.82498 | 8.947962192 | 0.997571554 | 612 | SFM | 5 | Foal |
| F8.6A | 26.17811 | 7.713807372 | 0.994518383 | 244 | SFM | 6 | Foal |
| F9.1A | 31.55301 | 8.200153296 | 0.995932398 | 375 | SFM | 1 | Foal |
| F9.2A | 44.84315 | 8.800315087 | 0.997338191 | 555 | SFM | 2 | Foal |
| F9.3A | 36.82586 | 8.553489396 | 0.996901936 | 456 | SFM | 3 | Foal |
| F9.4C | 64.5919 | 9.081638092 | 0.997799112 | 676 | SFM | 4 | Foal |
| F9.5B | 52.3463 | 8.749732294 | 0.997308575 | 520 | SFM | 5 | Foal |
| F9.6C | 61.9083 | 9.070553799 | 0.997844309 | 639 | SFM | 6 | Foal |

Results of t-tests for each alpha diversity comparing foals by week and dams**.** P-values less than 0.05 are highlighted.

| **Comparison** | **Shannon** | **Simpson** | **Observed_otus** | **PD_whole_tree** |
| --- | --- | --- | --- | --- |
| SF/D Foals week 1 | 0.472290998 | 0.72505751 | 0.249145157 | 0.540785358 |
| SF/D Foals week 2 | 0.584403459 | 0.4689303 | 0.055878172 | 0.095198656 |
| SF/D Foals week 3 | 0.329204335 | 0.74827054 | 0.105227714 | 0.7182498 |
| SF/D Foals week 4 | 0.082987195 | 0.11349893 | 0.158737357 | 0.506233986 |
| SF/D Foals week 5 | 0.493866577 | 0.32097032 | 0.503113421 | 0.169261481 |
| SF/D Foals week 6 | 0.610664923 | 0.56766609 | 0.913485112 | 0.750586385 |
| SF/D Dams | 0.848401282 | 0.71938578 | 0.978992248 | 0.083156913 |
| SF Foals/ Dams | 3.15254E-05 | 0.0110519 | 0.589073701 | 0.003913639 |
| D Foals/ Dams | 3.11051E-05 | 0.01600171 | 0.140846176 | 0.21783839 |
