# Supplementary material for: Development of the equine hindgut microbiome in semi-feral and domestic conventionally-managed foals
Source: Anim Microbiome. 2020 Nov 23;2:43. doi: 10.1186/s42523-020-00060-6 (PMC7807438; doi:10.1186/s42523-020-00060-6)
Supplement: Supplementary file 7 — Additional file 7. Significant ANOSIM and PERMANOVA comparisons. Statistical analysis of different foal and dam groups using ANOSIM and PERMANOVA tests. (note that foal gender was not found to be a significant factor). [file 42523_2020_60_MOESM7_ESM.docx]

Additional file 7

Statistical analysis of different foal and dam groups using ANOSIM and PERMANOVA tests. Only significant values shown. (note that foal gender was not found to be a significant factor)

|  | Group Comparison | Number of Groups Compared | Number of Subjects Compared | ANOSIM Significance Level | PERMANOVA Significance Level |
| --- | --- | --- | --- | --- | --- |
| Foals | SFM vs. DCM | 2 | 116 | p<0.01 | p<0.05 |
|  | Individual Foals | 20 | 116 | p<0.001 | p<0.001 |
|  | Weeks of Age | 6 | 116 | p<0.001 | p<0.001 |
|  | Grazing Access | 2 | 116 | p<0.05 | p<0.01 |
|  | Housing | 3 | 116 | p<0.01 | p<0.001 |
|  | DCM Age Week 1 vs. 2 | 2 | 20 | p<0.05 | p<0.05 |
|  | DCM Age Week 1 vs. 3 | 2 | 20 | p<0.01 | p<0.01 |
|  | DCM Age Week 1 vs. 4 | 2 | 20 | p<0.01 | p<0.01 |
|  | DCM Age Week 1 vs. 5 | 2 | 20 | p<0.01 | p<0.01 |
|  | DCM Age Week 1 vs. 6 | 2 | 16 | p<0.01 | p<0.01 |
|  | DCM Age Week 2 vs. 4 | 2 | 20 | p<0.01 | p<0.01 |
|  | DCM Age Week 2 vs. 5 | 2 | 20 | p<0.01 | p<0.01 |
|  | DCM Age Week 2 vs. 6 | 2 | 16 | p<0.01 | p<0.01 |
|  | SFM Age Week 1 vs. 2 | 2 | 20 | p<0.01 | p<0.05 |
|  | SFM Age Week 1 vs. 3 | 2 | 20 | p<0.01 | p<0.01 |
|  | SFM Age Week 1 vs. 4 | 2 | 20 | p<0.01 | p<0.01 |
|  | SFM Age Week 1 vs. 5 | 2 | 20 | p<0.01 | p<0.01 |
|  | SFM Age Week 1 vs. 6 | 2 | 20 | p<0.01 | p<0.01 |
|  | SFM Age Week 2 vs. 3 | 2 | 20 | p<0.05 | p<0.05 |
|  | SFM Age Week 2 vs. 4 | 2 | 20 | p<0.05 | p<0.05 |
|  | SFM Age Week 2 vs. 5 | 2 | 20 | p<0.01 | p<0.01 |
|  | SFM Age Week 2 vs. 6 | 2 | 20 | p<0.01 | p<0.01 |
|  | SFM Age Week 3 vs. 5 | 2 | 20 | p<0.05 | p<0.05 |
|  | SFM Age Week 3 vs. 6 | 2 | 20 | p<0.01 | p<0.05 |
| Dams | SFM vs. DCM Dams | 2 | 20 | p<0.01 | p<0.01 |
| FoalsDams | Foals vs. Dams | 2 | 136 | p<0.001 | p<0.001 |
|  | SFM vs. DCM All | 2 | 136 | p<0.05 | p<0.05 |
