# Supplementary material for: Development of the equine hindgut microbiome in semi-feral and domestic conventionally-managed foals
Source: Anim Microbiome. 2020 Nov 23;2:43. doi: 10.1186/s42523-020-00060-6 (PMC7807438; doi:10.1186/s42523-020-00060-6)
Supplement: Supplementary file 8 — Additional file 8. KEGG Functional categories. KEGG functions categorized into six different types of digestion. [file 42523_2020_60_MOESM8_ESM.docx]

Additional file 4:

KEGG functions categorized into six different types of digestion.

| **Type of Digestion** | **KEGG functions** |
| --- | --- |
| General carbohydrate | Carbohydrate digestion and absorption |
|  | Carbohydrate metabolism |
| Complex carbohydrate | Propanoate metabolism |
|  | Butanoate metabolism |
|  | Glycan biosynthesis and metabolism |
|  | Glycosaminoglycan degradation |
|  | Other glycan degradation |
| Simple carbohydrate | Fructose and mannose metabolism |
|  | Galactose metabolism |
| Starch | Starch and sucrose metabolism |
| Protein | Protein digestion and absorption |
|  | Amino acid metabolism |
|  | Alanine, aspartate and glutamate metabolism |
|  | Glycine, serine and threonine metabolism |
|  | Cysteine and methionine metabolism |
|  | Valine, leucine and isoleucine degradation |
|  | Lysine degradation |
|  | Arginine and proline metabolism |
|  | Histidine metabolism |
|  | Tyrosine metabolism |
|  | Phenylalanine metabolism |
|  | Tryptophan metabolism |
| Lipid | Glycerolipid metabolism |
|  | Glycerophospholipid metabolism |
|  | Lipid metabolism |
|  | Sphingolipid metabolism |
|  | Ether lipid metabolism |
|  | Fat digestion and absorption |
|  | Fatty acid metabolism |
